# Supplementary material for: A Likelihood Approach for Real-Time Calibration of Stochastic Compartmental Epidemic Models
Source: PLoS Comput Biol. 2017 Jan 17;13(1):e1005257. doi: 10.1371/journal.pcbi.1005257 (PMC5240920; doi:10.1371/journal.pcbi.1005257)
Supplement: S1 File — (TAR.GZ) [file pcbi.1005257.s014.tar.gz › HSPH_Online-SI-Revision/EnKF07/n1-severe/n1-severe_EnKF_table4.pdf]

1	R0	{1.51081, 13.7078}	{2.74407, 7.85468}	{{2.1657, 3.22613}, {3., 12.5987}}	{81.6293, 42.6992}	inf	60.	92.0472	{8.28631, 290.725}	139.867	0.428449	newly	28.	65.13	{7., 194.}	176.893	0.36826	Reff	0.861162	0.584537	{0.126745, 1.20255}	69.8304	ar	656.	1809.72	{1567., 2529.}	175.872
2	R0	{1.73182, 11.3873}	{2.58441, 7.53816}	{{1.18697, 1.81411}, {3., 17.9269}}	{62.826, 51.724}	inf	73.	68.6137	{10.4675, 94.6146}	75.7444	0.402313	newly	43.	95.41	{5., 137.}	188.488	0.471076	Reff	0.82781	0.496451	{0.0389624, 0.67086}	64.7045	ar	653.	1746.32	{1554., 2288.}	167.43
3	R0	{1.50449, 10.3474}	{2.75235, 6.37918}	{{2.49625, 3.53902}, {3., 6.52592}}	{87.383, 42.0922}	inf	61.	112.685	{6.98446, 220.715}	150.036	0.430548	newly	34.	108.47	{5., 348.}	257.676	0.43679	Reff	0.825965	0.669254	{0.252826, 1.62172}	60.9877	ar	651.	1906.19	{1616., 2697.}	192.81
4	R0	{1.63493, 9.1955}	{2.77332, 6.81876}	{{2.41366, 3.62739}, {3., 10.6491}}	{69.6295, 36.2491}	inf	51.	56.5122	{8.87109, 88.6207}	103.66	0.426739	newly	40.	55.13	{5., 122.}	129.925	0.476583	Reff	0.734084	0.481021	{0.130383, 1.06864}	61.2154	ar	663.	1751.03	{1599., 2025.}	164.107
5	R0	{1.53822, 8.05553}	{2.59944, 7.03504}	{{1.94938, 4.55816}, {3., 10.0277}}	{68.9902, 35.8354}	inf	25.	54.8933	{6.85988, 88.6262}	151.554	0.373164	newly	19.	69.08	{4., 109.}	298.105	0.466119	Reff	0.772186	0.620935	{0.114215, 1.9307}	72.0814	ar	621.	1777.12	{1570., 2431.}	186.171
6	R0	{1.69374, 17.0793}	{2.51467, 3.88446}	{{2.02325, 1.22454}, {3., 8.74358}}	{55.1442, 77.2563}	inf	95.	43.0951	{3.67601, 36.2594}	100.297	0.90454	newly	35.	46.67	{3., 70.}	133.286	0.556318	Reff	0.98745	0.450971	{0.0940353, 0.894311}	64.9586	ar	662.	1640.94	{1485., 2031.}	147.876
7	R0	{1.42179, 11.8031}	{2.49003, 6.49236}	{{2.046, 3.26212}, {3., 10.9124}}	{83.9249, 51.5296}	inf	48.	56.3436	{4.39177, 61.9526}	102.21	0.458169	newly	29.	58.13	{4., 70.}	164.103	0.451444	Reff	0.796202	0.419484	{0.0672459, 0.787043}	60.7068	ar	517.	1686.02	{1528., 2029.}	226.116
8	R0	{1.46683, 6.95875}	{2.75552, 5.5771}	{{2.38104, 0.772692}, {3., 6.93297}}	{87.8558, 44.2357}	inf	30.	49.1958	{5.48405, 44.7188}	131.297	0.353443	newly	30.	72.45	{4., 56.}	215.833	0.432388	Reff	0.545661	0.511376	{0.18832, 0.604223}	41.1509	ar	653.	1728.54	{1592., 1914.}	164.708
9	R0	{1.34525, 11.505}	{2.55492, 5.27691}	{{2.06392, 3.26462}, {3., 5.94796}}	{93.8738, 57.4929}	inf	49.	33.1537	{5.21628, 52.3755}	53.5734	0.395265	newly	38.	35.68	{4., 70.}	68.3158	0.40851	Reff	0.881139	0.457598	{0.109111, 0.880022}	57.4947	ar	532.	1638.4	{1521., 1841.}	207.97
10	R0	{1.77958, 14.372}	{2.62044, 9.9558}	{{2.05272, 5.95913}, {3., 14.796}}	{51.0304, 34.0388}	inf	58.	76.25	{10.2474, 80.6031}	128.693	0.459923	newly	28.	45.98	{6., 63.}	135.	0.397261	Reff	0.726069	0.561531	{0.127923, 0.947707}	54.4412	ar	671.	1712.32	{1487., 2339.}	155.189
11	R0	{1.60334, 19.9548}	{2.80711, 5.41733}	{{2.24199, 3.27291}, {3., 16.3355}}	{75.0789, 72.852}	inf	78.	32.1708	{4.63071, 34.529}	90.4873	0.793028	newly	24.	35.56	{3., 71.}	113.833	0.425606	Reff	0.928334	0.689056	{0.0428725, 2.05316}	65.7886	ar	617.	1649.72	{1488., 2247.}	167.378
12	R0	{1.68623, 7.58585}	{2.40599, 5.58373}	{{0.857949, 0.248449}, {3., 12.3318}}	{67.2614, 53.3171}	inf	21.	41.7482	{3.69035, 54.2182}	136.014	0.328532	newly	21.	61.5	{2., 78.}	242.286	0.432835	Reff	0.696413	0.447998	{0.0719867, 0.857091}	53.6315	ar	640.	1724.61	{1590., 1897.}	169.47
13	R0	{1.82689, 18.0022}	{2.52513, 4.44979}	{{2.05852, 1.09525}, {3., 9.96302}}	{47.1112, 75.4002}	inf	97.	21.7718	{3.37919, 18.0765}	87.1028	0.907155	newly	31.	30.62	{3., 39.}	92.9032	0.493427	Reff	0.811139	0.452702	{0.133275, 0.928282}	60.4104	ar	673.	1595.42	{1502., 1617.}	137.061
14	R0	{1.75911, 9.16883}	{2.33407, 6.25634}	{{0.853883, 0.51217}, {3., 8.70989}}	{60.0876, 40.5837}	inf	49.	57.4856	{4.77267, 93.3127}	107.076	0.481206	newly	35.	57.46	{2., 135.}	144.914	0.529306	Reff	0.809191	0.430876	{0.0716744, 0.814865}	57.5597	ar	661.	1738.19	{1583., 1951.}	162.964
15	R0	{1.46168, 11.3859}	{2.43942, 7.11173}	{{1.50699, 3.31225}, {3., 7.75594}}	{67.8739, 39.3756}	inf	42.	80.5782	{10.8968, 100.356}	132.72	0.273924	newly	28.	68.7	{8., 112.}	183.786	0.325328	Reff	0.69576	0.525216	{0.195815, 1.1125}	51.9678	ar	556.	1733.08	{1554., 2058.}	211.705
16	R0	{1.53577, 11.7581}	{2.3288, 6.26371}	{{0.865786, 3.02641}, {3., 11.8676}}	{75.1336, 51.9957}	inf	70.	73.2269	{3.74953, 88.7855}	117.659	0.601094	newly	40.	86.77	{2., 175.}	205.375	0.562788	Reff	0.7771	0.488996	{0.0927784, 0.862985}	56.1756	ar	625.	1781.75	{1586., 2174.}	185.08
17	R0	{1.70698, 9.72924}	{2.2784, 8.98607}	{{0.847079, 4.80016}, {3., 12.7675}}	{63.1908, 37.5512}	inf	45.	48.13	{5.61228, 107.809}	109.761	0.511222	newly	22.	37.38	{3., 83.}	151.545	0.47647	Reff	0.626462	0.464643	{0.0969997, 0.751706}	47.121	ar	675.	1740.54	{1574., 2302.}	157.858
18	R0	{1.5973, 14.931}	{2.49935, 5.93829}	{{0.940673, 1.20998}, {3., 9.50277}}	{72.8615, 60.8661}	inf	48.	38.2043	{6.393, 61.8267}	77.348	0.422867	newly	24.	44.77	{5., 90.}	141.958	0.384057	Reff	1.13089	0.470045	{0.0832334, 0.789673}	62.7259	ar	632.	1716.98	{1609., 1925.}	171.674
19	R0	{1.87468, 17.004}	{2.58231, 3.68683}	{{0.913672, 2.46796}, {3., 8.49621}}	{54.6526, 78.3179}	inf	76.	52.3504	{2.41299, 62.7644}	105.798	0.769354	newly	35.	81.53	{2., 214.}	218.486	0.565008	Reff	0.783616	0.449401	{0.104457, 0.991643}	63.5423	ar	692.	1666.83	{1496., 2038.}	140.871
20	R0	{1.84715, 12.531}	{2.11876, 5.62166}	{{0.144152, 2.3044}, {3., 8.56838}}	{69.4132, 55.138}	inf	47.	41.6818	{3.90621, 51.6592}	108.442	0.60344	newly	27.	34.29	{2., 44.}	120.926	0.498089	Reff	0.718541	0.336247	{0.0205126, 0.923291}	68.7288	ar	661.	1586.88	{1467., 1731.}	140.073
21	R0	{1.75938, 13.5959}	{2.28855, 10.0081}	{{0.892421, 3.79207}, {3., 15.8065}}	{59.6996, 39.2989}	inf	73.	45.5986	{4.5416, 72.3347}	94.9972	0.65334	newly	35.	28.98	{2., 58.}	96.7429	0.587865	Reff	0.932471	0.450326	{0.0689789, 0.765842}	59.0814	ar	620.	1765.65	{1590., 2338.}	184.782
22	R0	{1.63456, 10.7469}	{2.34891, 5.47849}	{{1.3907, 2.13659}, {3., 9.3022}}	{55.7008, 50.2998}	inf	44.	40.9699	{3.65629, 65.3024}	100.558	0.529115	newly	29.	44.95	{3., 91.}	136.241	0.466465	Reff	0.626036	0.527165	{0.0600411, 1.29493}	69.2981	ar	654.	1650.69	{1475., 2094.}	152.399
23	R0	{1.62702, 19.1392}	{2.79627, 5.84248}	{{2.50341, 2.54736}, {3., 9.66462}}	{72.2136, 69.4738}	inf	90.	63.3439	{1.76096, 95.6759}	98.042	0.744432	newly	31.	73.42	{1., 294.}	204.194	0.5521	Reff	0.810256	0.590426	{0.135539, 1.54531}	69.4611	ar	600.	1797.41	{1561., 2373.}	199.568
24	R0	{1.70466, 12.1202}	{2.58039, 3.76433}	{{1.13473, 2.91742}, {3., 6.892}}	{62.3681, 68.9417}	inf	45.	45.0952	{6.20421, 95.0592}	90.9735	0.435711	newly	23.	72.22	{8., 109.}	247.304	0.362149	Reff	0.758574	0.502234	{0.14894, 0.649293}	54.4876	ar	613.	1631.56	{1515., 1841.}	166.16
25	R0	{1.61735, 11.3169}	{2.74459, 5.5457}	{{1.86239, 3.19185}, {3., 5.2221}}	{69.6966, 56.5217}	inf	43.	24.0817	{4.98516, 46.2968}	59.1991	0.436111	newly	31.	29.95	{4., 51.}	83.1935	0.41517	Reff	0.608124	0.443989	{0.0404609, 0.550325}	51.4413	ar	681.	1565.65	{1458., 1810.}	129.905
26	R0	{1.56977, 12.3035}	{2.59443, 6.50737}	{{1.95483, 1.66726}, {3., 12.7162}}	{65.2748, 49.2237}	inf	36.	40.4893	{7.80286, 77.2454}	83.4143	0.355986	newly	18.	77.88	{6., 183.}	377.222	0.432089	Reff	0.846106	0.607163	{0.153361, 1.5966}	64.8292	ar	594.	1735.01	{1555., 2333.}	192.089
27	R0	{1.46317, 5.8298}	{2.54128, 9.4648}	{{1.25664, 6.033}, {3., 17.3028}}	{80.0162, 66.2915}	inf	14.	77.1249	{12.645, 125.636}	458.465	0.529449	newly	14.	58.95	{6., 93.}	344.5	0.43887	Reff	0.626237	0.477756	{0.0972718, 1.08576}	58.051	ar	586.	1734.26	{1571., 2094.}	195.949
28	R0	{1.70002, 14.9806}	{2.20253, 6.93123}	{{0.775458, 3.39674}, {3., 10.8797}}	{64.0169, 55.2279}	inf	101.	105.703	{9.96713, 175.784}	106.195	0.56054	newly	48.	89.77	{6., 212.}	155.521	0.493543	Reff	0.85511	0.46542	{0.0599295, 0.826165}	52.5706	ar	652.	1837.81	{1595., 2320.}	181.873
29	R0	{1.52729, 16.1014}	{2.24427, 11.1071}	{{0.336566, 7.48732}, {3., 16.3474}}	{89.8499, 31.5954}	inf	46.	36.8139	{3.31759, 63.5957}	65.6853	0.464548	newly	17.	23.64	{2., 31.}	104.471	0.424102	Reff	0.781972	0.359403	{0.0399527, 0.587634}	71.1748	ar	592.	1585.53	{1486., 1710.}	167.826
30	R0	{1.72795, 13.5635}	{2.37559, 5.28693}	{{1.37781, 3.31431}, {3., 9.13065}}	{53.0869, 61.8973}	inf	61.	72.504	{10.4205, 107.346}	95.4839	0.370807	newly	28.	61.96	{11., 102.}	151.714	0.334776	Reff	0.661805	0.414733	{0.184507, 0.781846}	51.2841	ar	668.	1766.13	{1578., 2174.}	164.391
31	R0	{1.51591, 18.1544}	{2.68301, 8.23456}	{{2.31902, 1.08769}, {3., 18.6496}}	{83.0275, 55.8916}	inf	51.	70.3428	{10.9064, 70.803}	93.5485	0.287537	newly	16.	74.22	{7., 93.}	382.5	0.412309	Reff	0.950476	0.497922	{0.0672136, 1.19829}	70.4301	ar	541.	1724.41	{1544., 2002.}	218.745
32	R0	{1.4282, 10.3534}	{2.51323, 3.43663}	{{1.22069, 1.00818}, {3., 8.26334}}	{82.6427, 68.5501}	inf	33.	47.3212	{3.32492, 122.148}	136.508	0.489797	newly	26.	76.76	{3., 152.}	258.923	0.443651	Reff	1.03687	0.460851	{0.111483, 0.694354}	64.4257	ar	521.	1673.97	{1537., 2149.}	221.299
33	R0	{1.56693, 11.3972}	{2.40491, 7.58627}	{{0.916524, 3.57895}, {3., 13.805}}	{72.7628, 43.0521}	inf	84.	31.589	{6.87058, 45.9617}	79.0553	0.685378	newly	50.	35.65	{5., 68.}	97.62	0.652921	Reff	0.634607	0.535993	{0.0967235, 1.02414}	52.6849	ar	695.	1703.64	{1570., 1986.}	145.128
34	R0	{1.50675, 19.8823}	{2.83745, 4.6385}	{{2.30883, 0.214194}, {3., 16.5514}}	{88.3158, 76.6702}	inf	63.	50.3319	{7.30008, 139.061}	90.6908	0.555866	newly	29.	76.01	{5., 203.}	207.759	0.408249	Reff	0.797071	0.563534	{0.0993724, 1.37285}	72.0347	ar	587.	1693.22	{1509., 2226.}	188.453
35	R0	{1.72889, 7.76068}	{2.22729, 7.78918}	{{0.817364, 4.72185}, {3., 10.7172}}	{61.4843, 34.4554}	inf	43.	57.0126	{6.6574, 106.536}	121.985	0.463924	newly	34.	47.27	{4., 105.}	135.5	0.519869	Reff	0.7002	0.461601	{0.0600213, 0.744674}	52.9685	ar	650.	1767.99	{1584., 2375.}	171.998
36	R0	{1.62644, 4.12303}	{2.63057, 5.02278}	{{1.86961, 3.67171}, {3., 5.29009}}	{63.915, 27.9038}	inf	8.	45.7775	{5.80902, 49.1293}	487.778	0.519754	newly	9.	66.97	{5., 50.}	666.111	0.52626	Reff	0.57088	0.515239	{0.197244, 0.604411}	42.1217	ar	652.	1758.47	{1590., 2007.}	169.704
37	R0	{1.70878, 15.8072}	{2.77628, 4.73272}	{{2.27984, 3.29562}, {3., 6.18233}}	{70.2372, 70.0597}	inf	45.	42.4586	{4.78495, 39.0737}	95.0927	0.492519	newly	13.	74.19	{5., 59.}	502.077	0.482402	Reff	0.772369	0.594104	{0.10771, 1.55105}	71.6713	ar	674.	1631.32	{1470., 2050.}	142.036
38	R0	{1.58967, 6.80568}	{2.78819, 5.92603}	{{2.40154, 1.5195}, {3., 6.87408}}	{75.3944, 34.2134}	inf	25.	40.1792	{2.40271, 60.6868}	117.244	0.369397	newly	22.	61.07	{2., 61.}	234.773	0.44161	Reff	0.661303	0.588973	{0.280732, 1.10615}	49.9973	ar	610.	1746.01	{1603., 1895.}	186.231
39	R0	{1.344, 8.19498}	{2.57066, 3.05113}	{{2.1017, 0.788006}, {3., 4.47352}}	{96.2701, 63.1834}	inf	37.	24.6348	{3.70116, 25.9128}	87.3768	0.574002	newly	28.	35.27	{4., 45.}	111.25	0.441078	Reff	0.756672	0.407941	{0.129134, 0.794577}	53.4697	ar	551.	1600.3	{1522., 1612.}	190.436
40	R0	{1.61509, 7.90825}	{2.62315, 8.49664}	{{1.82494, 4.70095}, {3., 16.6335}}	{68.7388, 53.6818}	inf	33.	30.8756	{6.26488, 47.4428}	46.1101	0.282045	newly	23.	22.84	{4., 36.}	59.0435	0.328276	Reff	0.814005	0.407167	{0.094771, 0.597076}	52.2072	ar	581.	1735.01	{1636., 1800.}	198.625
41	R0	{1.60017, 5.90605}	{2.60136, 7.16202}	{{1.94418, 3.36314}, {3., 9.61975}}	{62.5675, 44.3944}	inf	23.	59.3438	{7.22496, 91.9842}	199.77	0.401654	newly	19.	66.05	{4., 166.}	300.474	0.503543	Reff	0.611265	0.598737	{0.104906, 1.7763}	69.7667	ar	661.	1792.52	{1567., 2494.}	171.183
42	R0	{1.64436, 15.1337}	{2.61907, 5.09097}	{{1.95978, 1.73434}, {3., 9.32322}}	{59.2757, 66.36}	inf	68.	59.8011	{6.12973, 86.1177}	81.06	0.463028	newly	31.	67.67	{5., 134.}	166.484	0.427884	Reff	0.902754	0.501873	{0.0905887, 1.31962}	65.4501	ar	640.	1746.81	{1583., 1990.}	172.939
43	R0	{1.48917, 11.917}	{2.66899, 2.65978}	{{1.63386, 1.56014}, {3., 3.24423}}	{79.2269, 77.6808}	inf	22.	21.0729	{1.92118, 17.1662}	68.1622	0.348913	newly	12.	43.44	{3., 37.}	291.833	0.44318	Reff	0.814576	0.517436	{0.190342, 0.679283}	53.8559	ar	551.	1536.23	{1451., 1640.}	178.808
44	R0	{1.65577, 7.89317}	{2.79805, 6.53123}	{{2.42, 0.46878}, {3., 7.51998}}	{68.9876, 32.2728}	inf	38.	41.1614	{6.35298, 57.5724}	88.1467	0.388257	newly	25.	42.5	{4., 55.}	135.44	0.404311	Reff	0.819606	0.456019	{0.183624, 0.650713}	54.9377	ar	576.	1726.85	{1600., 1941.}	199.8
45	R0	{1.6909, 12.3275}	{2.57149, 9.89261}	{{1.51158, 5.67349}, {3., 17.7608}}	{69.0907, 39.7384}	inf	39.	73.5307	{9.50003, 80.1877}	125.74	0.33988	newly	24.	51.34	{5., 53.}	159.667	0.379386	Reff	0.749069	0.475157	{0.0852849, 0.77327}	51.8565	ar	675.	1777.4	{1566., 2259.}	163.319
46	R0	{1.62351, 16.0698}	{2.61281, 3.22066}	{{2.08101, 1.62582}, {3., 6.38367}}	{62.3863, 80.0665}	inf	55.	39.8947	{4.73883, 62.1994}	79.2606	0.518147	newly	26.	76.32	{6., 96.}	241.154	0.421135	Reff	1.091	0.507935	{0.195013, 0.788108}	64.2949	ar	591.	1669.72	{1534., 1912.}	182.525
47	R0	{1.52372, 4.38599}	{2.60714, 5.97376}	{{1.7642, 1.4405}, {3., 8.52904}}	{72.917, 57.7429}	inf	12.	31.6308	{3.46286, 65.1944}	190.019	0.425681	newly	15.	39.7	{3., 69.}	204.4	0.429767	Reff	0.594251	0.528047	{0.144831, 0.878827}	53.0331	ar	618.	1752.31	{1601., 2267.}	183.545
48	R0	{1.44306, 11.788}	{2.69531, 8.43332}	{{2.41183, 6.08134}, {3., 12.3098}}	{91.34, 31.2412}	inf	18.	56.1946	{8.53512, 105.186}	235.769	0.434398	newly	11.	51.02	{5., 88.}	386.364	0.489989	Reff	0.793683	0.556656	{0.0965921, 1.19646}	67.665	ar	534.	1744.51	{1536., 2299.}	226.687
49	R0	{1.63624, 11.3893}	{2.79634, 6.30537}	{{2.41675, 0.773821}, {3., 7.66585}}	{70.9004, 50.4304}	inf	48.	40.3127	{10.0174, 68.9765}	84.7831	0.428331	newly	31.	59.97	{8., 113.}	175.645	0.433447	Reff	0.713401	0.526588	{0.173722, 0.962689}	58.983	ar	643.	1721.17	{1590., 2117.}	167.678
50	R0	{1.46113, 11.1322}	{2.51159, 4.15872}	{{1.19084, 1.03646}, {3., 11.504}}	{80.9903, 63.7284}	inf	30.	44.8727	{3.46306, 70.1711}	117.748	0.39109	newly	18.	48.32	{2., 81.}	212.889	0.451697	Reff	0.936584	0.418349	{0.10744, 0.696164}	58.4942	ar	501.	1671.4	{1542., 1979.}	233.613
